# Neural Regulation of Vascular Development: Molecular Mechanisms and Interactions

**DOI:** 10.3390/biom14080966

**Published:** 2024-08-08

**Authors:** Yu Zhang, Xinyu Shen, Shunze Deng, Qiurong Chen, Bing Xu

**Affiliations:** School of Life Sciences, Nantong University, Nantong 226019, China

**Keywords:** blood vessel, central nervous system, brain vascular development, neurovascular unit, blood–brain barrier

## Abstract

As a critical part of the circulatory system, blood vessels transport oxygen and nutrients to every corner of the body, nourishing each cell, and also remove waste and toxins. Defects in vascular development and function are closely associated with many diseases, such as heart disease, stroke, and atherosclerosis. In the nervous system, the nervous and vascular systems are intricately connected in both development and function. First, peripheral blood vessels and nerves exhibit parallel distribution patterns. In the central nervous system (CNS), nerves and blood vessels form a complex interface known as the neurovascular unit. Second, the vascular system employs similar cellular and molecular mechanisms as the nervous system for its development. Third, the development and function of CNS vasculature are tightly regulated by CNS-specific signaling pathways and neural activity. Additionally, vascular endothelial cells within the CNS are tightly connected and interact with pericytes, astrocytes, neurons, and microglia to form the blood–brain barrier (BBB). The BBB strictly controls material exchanges between the blood and brain, maintaining the brain’s microenvironmental homeostasis, which is crucial for the normal development and function of the CNS. Here, we comprehensively summarize research on neural regulation of vascular and BBB development and propose directions for future research.

## 1. Introduction

An intricate and complex vascular network is responsible for delivering oxygen, nutrients, and signaling molecules to various tissues and organs, while removing cellular metabolic byproducts, thereby ensuring normal development and functional maintenance of the body [1]. Abnormalities in the development and function of blood vessels are related to a variety of diseases, including cardiovascular diseases, stroke, and atherosclerosis, etc. [2] As the control center of human body, the brain, although comprising only 2% of body weight, consumes nearly 20% of the body’s energy, including total oxygen and glucose [3]. If blood flow ceases, the brain will be subject to irreversible damage due to ischemia within just a few minutes. All of these results demonstrate the crucial roles of central nervous system (CNS) vasculature in the normal development and functions of the brain. The complex brain vascular network originates from the perineural vascular plexus (PNVP) through sprouting angiogenesis, which is controlled by various factors secreted from neural progenitor cells, including vascular endothelial growth factor (VEGF), Wnt, etc. [4,5]

Different from the peripheral vascular system, the CNS-invading endothelial cells tightly connect with each other through tight and adherens junction proteins and recruit different perivascular cells, including pericytes and astrocytes, to form the blood–brain barrier (BBB) [6,7]. The BBB strictly controls the material exchanges between blood vessels and brain parenchyma to maintain the hemostasis of brain microenvironment, which is critical for the normal development and function of the brain [6]. Furthermore, the neurovascular unit (NVU) formed by endothelial cells and perivascular cells, including pericytes, astrocytes, neurons, and microglia, is the functional unit between endothelial cells and different brain cells, which also regulates the CNS vascular developments [8]. The defects of CNS vascular development and BBB are closely associated with various neurological diseases, including stroke, arteriovenous malformations, cavernous angiomas, and neurodegenerative diseases [9].

The development of the CNS vascular network is regulated not only by classical vascular developmental molecular mechanisms, such as VEGF, but also by CNS-specific signaling pathways, including Wnt, Gpr124, NogoA, etc. [7,8] Additionally, as the specific properties of the CNS, neural activity plays fundamental roles in the development of CNS vasculature and BBB [8]. However, the underlying mechanisms are still not fully understood. In this review, we provide a comprehensive summary of the current understanding of the interplay between the nervous and vascular systems, including the similarities between the nervous and vascular systems, CNS-specific regulation of brain vasculature development, and new directions for future studies to achieve a deeper understanding of neurovascular development.

## 2. Similarity between Nervous and Vascular Systems

### 2.1. Neurovascular Congruency

In 1543, by dissecting human bodies, the anatomist Andreas Vesalius first described the similarities in the branching patterns of nerves and blood vessels at the macroscopic level, which laid the foundation for the study of neurovascular links [10]. With the rapid development of modern biological methods, scientists have observed the parallel growth of peripheral blood vessels and nerve fibers, a phenomenon known as neurovascular congruency [11,12,13]. For example, in the developing mouse skin, the nerves and arterial vessels show parallel growth [11]. In mouse whisker follicles, nerve and blood vessels are organized into a double-ring structure [13]. In zebrafish trunks, the motoneuron axons grow along the dorsal aorta [14]. So, what are the fundamental principles behind the parallel establishment of nerves and blood vessels?

**Figure 1:**
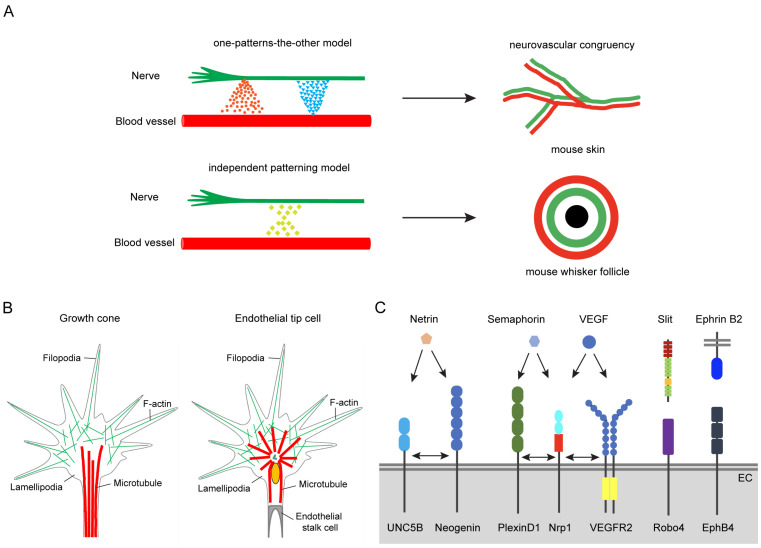
Similarity between the nervous and vascular systems. (**A**). Two models of neurovascular congruency. In the one-patterns-the-other model, the nerves and blood vessels secrete different factors that mutually guide their growth. In the independent patterning model, the nerves and blood vessels respond to the same signals, but grow independently, finally forming the neurovascular congruency according to the gradient of these factors. (**B**). Similar cellular structures of axons and endothelial cells during directional growth. Both the growth cone (specialized structure of axonal terminal) and the endothelial tip cell (specialized endothelial cell) contain actin-enriched filopodia to explore the surrounding microenvironment. They can grow or retract according to attractive or repulsive cues, finally guiding the correct growth of axons or blood vessels. (**C**). The functions of four classical axon guidance cues in endothelial cells. Through different receptors, axon guidance cues exert different functions on the vascular development.

After years of research, two models are proposed to explain this phenomenon of neurovascular congruency: the one-patterns-the-other model and the independent patterning model [3] (Figure 1A). In the one-patterns-the-other model, the growth of blood vessels and nerves is not entirely independent but mutually guided. For example, blood vessels direct the neuronal development and attract axons to grow along the blood vessels via secreting classic angiogenic factors, such as VEGF, FGF2, artemin, endothelin-3, and Neurotrophin-3 [15,16]. Conversely, VEGF and CXCL12 released by neurons also attract blood vessels to grow along nerve fibers [11,12]. This mutual guidance mechanism ensures the nerves and blood vessels display parallel growth (Figure 1A). In the independent patterning model, although blood vessels and nerves grow in parallel, they each respond to common guidance signals for growth instead of mutual guidance, resulting in a fixed neurovascular congruency arrangement [13] (Figure 1A). For instance, during the development of mouse whisker follicles, an internal nerve ring forms around each hair follicle, followed by an external vascular ring, creating a fixed double-ring structure. Further studies demonstrated in mutant mice lacking trigeminal neurons that although the formation of the nerve ring is affected, the vascular ring develops normally; conversely, in mice with abnormal vascular rings, the nerve ring remains unaffected [13]. These results suggest that nerves and blood vessels possess independent developmental mechanisms that can respond to common guidance signals to form a neurovascular congruency.

### 2.2. Cellular and Molecular Similarity

The existence of such a precise organization between nervous and vascular systems suggests that they may share common cellular and molecular mechanisms during development.

#### 2.2.1. Cellular Similarity 

In 1890, neurobiologist Santiago Ramón y Cajal first discovered a specialized cellular structure at the leading edge of growing axons, which he named the growth cone [17]. These growth cones are composed of hand-shaped, actin-rich lamellipodia and filopodia, which dynamically extend and retract through sensing the attractive and repulsive signal molecules in the local microenvironment, thereby guiding the axon along the correct path (Figure 1B). Upon reaching the target, unnecessary axon branches are pruned to precisely establish the neural projection pattern [18,19].

More than a century later, Gerhardt et al. discovered that the process of angiogenesis bears a remarkable similarity to the guidance mechanisms of neural growth cones [20]. With the developing retinal vessels as a model, they found the leading endothelial cell specialize into a structure that resembles the axonal growth cone, named endothelial tip cells (ETCs) [20] (Figure 1B). ETCs are also actin-rich and form lamellipodia and filopodia, which dynamically extend and retract via sensing attractive or repulsive signals in the local environment, guiding the forward growth of blood vessels and precisely targeting the correct locations [7,21].

#### 2.2.2. Molecular Similarity

The nervous and vascular systems not only display similar cellular structures, but also use similar molecular factors to guide their growth. For example, they use the four classical axonal guidance cue families: netrins, slits, semaphorins, and ephrins, and other molecules (Figure 1C), including wingless/integrated (Wnt), Sonic hedgehog (Shh), bone morphogenetic proteins (BMPs), Nogo-A, and Nogo-B [8,22].

Netrin proteins are secreted guidance cues, acting as attractive or repulsive effects on axon growth through different receptors [23]. Specifically, through deleted in colorectal cancer (DCC) receptors, netrin exerts an attractive effect and promotes axon growth along the netrin gradient [24,25]; whereas through the uncoordinated 5 (UNC5) receptor, netrin exerts a repulsive effect [26,27]. In the vascular system, endothelial cells express the UNC5 receptor but not the DCC receptor [22]. Loss of function of UNC5B increases in vascular branching and ETC filopodia [28]. Through UNC5B, netrin-1 acts as an anti-angiogenic factor to inhibit vascular development [29]. By binding to a novel CD146 receptor, netrin-1 acts as a pro-angiogenic factor to promote vascular development [30]. By binding to neogenin and recruiting UNC5B, netrin-4 inhibits angiogenesis and vascular development [31]. Netrin-4 can also promote angiogenesis via protein kinase signaling pathways [32]. In summary, netrin has a bidirectional regulatory function in the vascular development, similar to that in axon guidance.

Slit proteins are secreted guidance cues that exert a repulsive effect on axon growth by binding to the roundabout (Robo) receptors [33,34,35]. In mammals, the Robo family contains Robo1–4, with Robo4 being specifically expressed in vascular endothelial cells [36]. Through negatively regulating the VEGF signaling pathway, Robo4 maintains vascular stability and inhibits angiogenesis [37]. Through Robo4, Slit2 maintains the vascular stability via inhibiting ARF6 and Rac [38,39]. In Robo4 knockout mice, retinal vascular permeability significantly increases, and excessive vascular formation occurs during induced pathological angiogenesis [38]. Recent studies also found that Robo4 can bind and activate UNC5B to inhibit angiogenesis [40].

Semaphorins are a family of secreted or transmembrane proteins that primarily inhibit the axonal growth by binding to specific receptors, such as Plexin or Neuropilin (Nrp) [41]. In vascular development, most of the studies are focused on the roles of the Semaphorin3 (Sema3) family and their receptors, PlexinD1 and Nrp. The Sema3 family comprises seven members, Sema3A to Sema3G. Most of them bind to Nrp, acting as co-receptors for Plexin, except for Sema3E, which directly binds to the PlexinD1 receptor without involving Nrp [42,43]. PlexinD1 is primarily expressed in endothelial cells and its critical role in angiogenesis has been extensively studied. In the *PlexinD1* knockout mouse models, vascular development shows significant increased angiogenic sprouting [44,45]. In the development of retinal vasculature, Sema3E-PlexinD1 negatively regulates the angiogenesis via counteracting the VEGF-induced Delta-like 4 (Dll4)-Notch signaling to inhibit the ETC and stalk cell selection [46]. In the mouse model of ischemic retinopathy, Sema3E-PlexinD1 activates the small GTPase RhoJ, preventing VEGF-induced filopodia disorientated projection [47]. Sema3A also inhibits the angiogenesis and vascular development via Nrp1 and PlexinA-PlexinD1 receptor complex [48]. However, because of the normal vascular development in *Sema3a* knockout and *Nrp1* mutant (lack the Sema-binding domain) mice, the exact role of Sema3A in CNS vascular development is still unknown [49,50]. In zebrafish models with mutated *PlexinD1*, intersegmental vessels exhibit premature sprouting and abnormal branching [51]. Sema3A-PlexinD1 inhibits angiogenesis by promoting the expression and release of soluble VEGF receptor (sFlt), thereby inhibiting the VEGF signaling pathway [52].

Eph receptors can be divided into two subfamilies: EphA and EphB, with two kinds of ligands: ephrinA and ephrinB [53]. The interaction between ephrin and Eph plays a bidirectional regulatory role in the nervous and vascular systems, with both attractive and repulsive effects [54]. The specific expression of ephrinB2 and its receptor EphB4 in arteries or veins is particularly notable [55]. EphrinB2 is specifically expressed in arterial angioblasts, while EphB4 is specifically expressed in veins, suggesting ephrinB2/EphB4 is crucial for the development and maintenance of arteriovenous structures [56]. Mechanistically, ephrinB2 promotes the extension of ETC filopodia and angiogenic sprouting by activating the VEGF signaling pathway [57,58]. In mutant mouse models with a disrupted intracellular PDZ domain of ephrinB2, ETC numbers and filopodial extension ability significantly decrease, leading to reduced angiogenic sprouting [59].

## 3. CNS Vascular Development and Specific Molecular Mechanisms

### 3.1. CNS Vascular Development

The formation of the CNS vascular network is a complex and intricate process. During the mouse brain vascular development, a perineural vascular plexus (PNVP) derived from the mesoderm form around the neural tube via vasculogenesis at embryonic day 8.5 (E8.5), which establishes the basis for arteries and veins of the pia and arachnoid mater. Subsequently, at E9.5, the endothelial cells from the PNVP invade CNS parenchyma through sprouting angiogenesis, growing towards the ventricles to form the intraneural vascular plexus (INVP) [7,17,60]. Once the INVP are inside the ventricular zone, they branch in a circumferential fashion parallel to the ependyma, giving rise to a periventricular vascular plexus [61]. After that, this initial vascular network is further expanded and matured through additional sprouting angiogenesis and remodeling processes. The invading growth of endothelial cells from PNVP into the brain is mainly instructed by neural tube-derived VEGF [62].

During CNS vascular development, the invading endothelial cells start specialized according to the CNS microenvironment, including (1) tighter connections between these endothelial cells, greatly restricting free diffusion, paracellular transportation, and transcytosis between blood and the brain; (2) more specific transporters for specific and efficient directional transportation of brain-needed compounds or xenobiotic compounds; and (3) extremely low expression of leukocyte adhesion molecules (LAMs), limiting the entry of peripheral immune cells into the CNS, thereby preventing inflammation-related damage to brain tissue [63,64]. Furthermore, when the endothelial cells invade the brain parenchyma, they recruit pericytes and astrocytes, and intimately connect with neurons and microglia to form the BBB. The BBB strictly controls the material exchange between blood and the brain to maintain the homeostasis of the brain microenvironment, which is vital for the normal development and function of the CNS [6].

### 3.2. CNS-Specific Molecular Mechanisms during Vascular Development

During CNS vascular development, many general vascular development-related signaling pathways participate in this process, including VEGF, Notch, Hippo, angiopoietin-Tie1/2, TGFβ, retinoic acid (RA), and the abovementioned classical axon guidance cues [65,66,67,68]. Apart from these general molecular mechanisms, some CNS-specific molecular mechanisms were found, such as Wnt, Gpr124, DR6/TROY, Mfsd2a, Ppil4, etc. [5,8].

#### 3.2.1. Wnt/β-Catenin Signaling Pathway

The Wnt/β-catenin signaling pathway is crucial for CNS vascular development and the establishment of a functional BBB [69]. During mouse development, neural progenitor cells in the forebrain and ventricular regions specifically express *Wnt7a* and *Wnt7b*, while those in the hindbrain and dorsal spinal cord express *Wnt1*, *Wnt3a*, and *Wnt3b* [70,71]. These proteins activate the canonical Wnt signaling pathway by binding to frizzled receptors on vascular endothelial cells, inhibiting β-catenin degradation, and subsequently activating genes related to vascular development. In embryonic CNS endothelial cells, Wnt/β-catenin signaling is specifically activated [71]. In Wnt7a/b knockout mice, brain vascular development is severely impaired and the expression of BBB-related genes, e.g., glucose transporter-1 (Glut-1) and Claudin-3, are decreased, leading to severe intracerebral hemorrhages and embryonic death [71]. Similarly, the specific deletion of β-catenin in vascular endothelial cells results in abnormal CNS vascular development and BBB defects, without affecting peripheral blood vessels [70,71].

High-resolution in vivo imaging studies have shown that Wnt/β-catenin signaling promotes the formation of endothelial cell junctions during brain vascularization by inhibiting Sphingosine-1-phosphate receptors (S1prs) and upregulating VE-cadherin expression, which facilitates BBB maturation and VE-cadherin stability [72]. Additionally, the transforming growth factor-β (TGF-β) family member Norrin can bind to frizzled-4, activating the canonical Wnt signaling pathway and regulating retinal vascular development [73,74]. Mice lacking Norrin or frizzled-4 exhibit abnormal retinal vascularization and BBB leakage, highlighting the importance of frizzled-4 signaling in maintaining retinal and BBB integrity [74]. Screening for genes specifically expressed in brain endothelial cells revealed that death receptors DR6 and TROY are enriched in CNS endothelial cells and play crucial roles in CNS vascularization and BBB formation [75]. Their expression is regulated by the Wnt/β-catenin pathway and is essential for VEGF downstream signaling, suggesting an interaction between Wnt and VEGF pathways in regulating brain vasculature and BBB development. In zebrafish, as the downstream of Wnt signaling pathway, *ppil4* mutation causes necrosis in the dorsal midbrain and embryonic lethality [76]. Because of the crucial roles of Wnt/β-catenin signaling in brain angiogenesis and BBB development, engineered Wnt ligands were developed to repair BBB damage in neurological diseases, offering new therapeutic strategies [77].

However, the complexity of Wnt/β-catenin signaling means its disruption leads to widespread CNS vascular abnormalities, complicating the distinction between its specific roles in angiogenesis and BBB development. Recent studies found Wnt/β-catenin signaling regulates the expression of metalloproteinase MMP25 in brain endothelial cells, which degrades collagen IV α5/6 secreted by meningeal fibroblasts, promoting the infiltration of endothelial cell into the brain parenchyma. When meningeal basement membrane is impaired, the Wnt-deficient endothelial cells can still invade the brain, but BBB development is impaired, indicating that CNS angiogenesis and BBB formation are closely related yet distinct biological events [78]. This finding provides new insights into the role of Wnt/β-catenin signaling in CNS vascular development.

#### 3.2.2. Gpr124

The orphan G protein-coupled receptor 124 (Gpr124) is highly expressed in CNS vascular endothelial cells and pericytes, and specifically regulates brain vascular and BBB development [79,80]. Both systemic and endothelial cell-specific knockouts of *Gpr124* result in severe abnormalities in brain vascular development, leading to significant hemorrhages in the forebrain and ventral spinal cord, ultimately causing embryonic death [79]. Endothelial overexpression of Gpr124 leads to CNS-specific hyperproliferative vascular malformation [79]. Downstream studies show the intracellular domain of Gpr124 is not necessary for brain vascular and BBB development. Instead, Gpr124 functions mainly by binding to RECK, stabilizing newly synthesized Wnt proteins, and activating the downstream β-catenin signaling pathway via frizzled receptors, thereby regulating brain vascular and BBB development [81,82,83,84]. Recent studies further explored the role of Gpr124 in adult mice and found the specific deletion of *Gpr124* in adult mice endothelial cells did not affect CNS angiogenesis and BBB development under normal conditions. However, under pathological conditions, for example ischemic stroke and glioblastoma, Gpr124 is necessary for the activation of Wnt signaling pathway and the maintenance of BBB integrity [85]. This finding suggests that Gpr124 may play a different role in maintaining brain vascular homeostasis in adult mice, particularly in response to pathological challenges.

#### 3.2.3. Mfsd2a

The major facilitator domain-containing protein 2a (Mfsd2a) is a transmembrane protein receptor belonging to the major facilitator superfamily [86]. These receptors primarily use concentration gradients to drive transmembrane transport. *Mfsd2a* is specifically expressed in CNS vascular endothelial cells, highlighting its crucial role in CNS vascular and BBB development. Studies show Mfsd2a promotes BBB stability and function by reducing endocytosis, thereby minimizing the entry of unnecessary substances into the brain [87]. In mice with *Mfsd2a* deletions in endothelial cells, the CNS vasculature remains normal, but the BBB displays increased permeability. Further study demonstrated the endocytosis of BBB is significantly increased under *Mfsd2a* knockout conditions, similar to the phenotype observed with pericyte loss [87,88,89]. Correspondingly, in mouse models lacking pericytes, Mfsd2a expression levels are significantly reduced, suggesting a potential collaborative mechanism between Mfsd2a and pericytes in regulating the BBB development and maintenance. Studies on its downstream mechanisms have shown that Mfsd2a interacts with Spinster homolog 2 (Spns2) to regulate the efflux of sphingosine-1-phosphate (S1P) in brain endothelial cells, thus playing a role in the formation and maintenance of the BBB [90].

## 4. Neural Regulation of CNS Vascular Development and Function

### 4.1. Regulation of CNS Vascular Development by Neural Progenitor Cells

**Figure 2:**
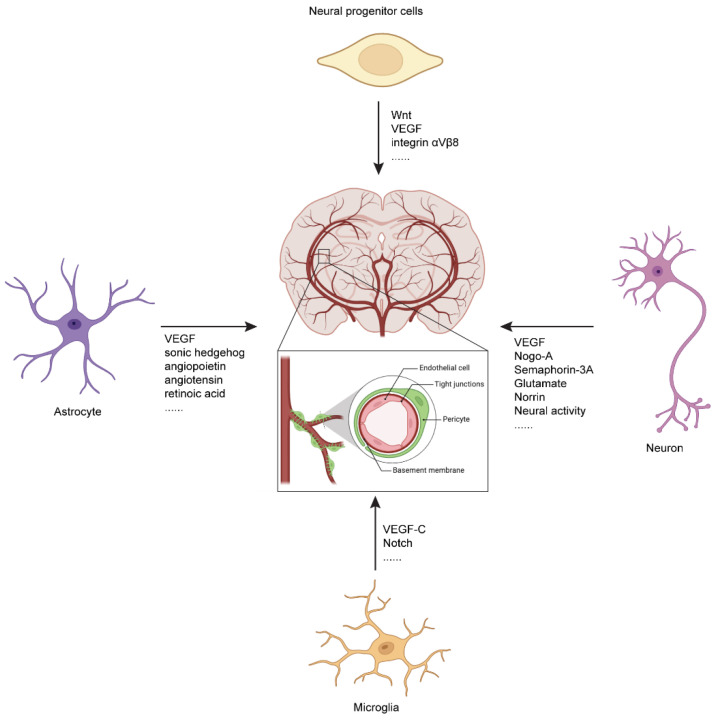
Neural regulation of CNS vascular and BBB development. Different cell types in the CNS regulate CNS vascular development and BBB permeability by secreting different factors. Neural activity can also affect CNS vascular development and BBB permeability according to the intensity of neural activity and developmental period. NPCs regulate brain vascular development via secreting different angiogenic factors, for example Wnt, VEGF, etc. The integrin αVβ8 in NPCs is also critical for brain vascular development. Astrocytes mainly regulate BBB development and maintenance via secreting a variety of factors, including VEGF, Shh, angiopoietin, angiotensin, RA, etc. Microglia also can regulate the brain vascular development and repairment via VEGF-C, and Notch signaling pathways. Neurons can regulate brain vascular development and function through secreting different factors, including VEGF, Nogo-A, Semaphorin-3A, glutamate, Norrin, etc. Furthermore, neural activity also plays important roles in brain vascular and BBB development and function.

Neural progenitor cells (NPCs) regulate CNS vascular development through various molecules (Table 1). During embryonic development, NPCs in the subventricular zone secrete VEGF, guiding the infiltration of new blood vessels from PNVP into the brain parenchyma [62,91]. Specific knockout of VEGF in NPCs results in a significant reduction in CNS vascular density, severe vascular abnormalities, and subsequent neonatal death. Wnt secreted by NPCs is crucial for CNS vascular development, Glut1 expression, and BBB development and maintenance [69]. Additionally, integrin αVβ8 in NPCs is involved in CNS vascular development. Specific knockout of integrin αVβ8 in NPCs leads to abnormal brain vascular development and hemorrhage [68]. In zebrafish models, the loss of NPCs results in excessive sprouting of perispinal blood vessels and the formation of ectopic branches [92] (Figure 2).

### 4.2. Regulation of CNS Vascular Development by Neurons

Neurons secrete various factors to regulate CNS vascular development and function (Table 1). For example, specific deletion of VEGF signaling in retinal interneurons leads to abnormal retinal vascular development [93]. Neuronal expression of the axonal growth inhibitory membrane protein Nogo-A negatively regulates CNS angiogenesis [94]. Our research has shown that neurons release exosomes containing *miR-132*, which promote brain vascular development and BBB maintenance in zebrafish [95]. Recent studies have found that during retinal vascular development, a group of *FAM19a4/Nts*-positive retinal ganglion cells, which directly contact retinal vessels, regulate the construction of the retinal three-dimensional vascular network through Piezo2 [96]. Under pathological conditions, retinal ganglion cells release Sema3A which inhibits vascular reconstruction and disrupts the blood–retina barrier (BRB) in ischemic retina [97] (Figure 2).

### 4.3. Regulation of CNS Vascular Development by Astrocytes

Astrocytes secrete various factors to regulate BBB integrity [98] (Table 1). For instance, astrocyte-secreted Shh promotes the expression of tight junction proteins occludin and Claudin-5 in CNS endothelial cells, while inhibiting the expression of chemokines and cell adhesion molecules, thereby maintaining BBB function [99]. Specific knockout of the Shh downstream gene smoothened in endothelial cells leads to reduced expression of tight junction proteins, causing plasma protein leakage from brain vessels [99]. The angiogenic factors like angiopoietin (Ang) and angiotensin secreted by astrocytes can promote the formation of tight junctions in endothelial cells, further regulating BBB function [100]. Astrocyte-derived retinoic acid (RA) upregulates the expression of junction proteins and transporters, promoting BBB development [101]. Apolipoprotein E (APOE) expressed by astrocytes binds to low-density lipoprotein receptor-related protein-1 (LRP-1) on pericytes or endothelial cells, regulating the expression of matrix metalloproteinase-9 (MMP-9) and BBB function [102]. Additionally, astrocytic end-feet are enriched in aquaporin-4 (Aqp-4) and Kir 4.1, which play crucial roles in maintaining brain water and ion homeostasis [103,104]. Under inflammatory conditions, astrocytes secrete VEGF which leads to increased BBB permeability [105]. Astrocytes are also in close contact with neurons and brain microvessels, serving as critical mediators of neurovascular interactions [106] (Figure 2).

### 4.4. Regulation of CNS Vascular Development by Microglia

Microglia are CNS resident immune cells which play crucial roles in retinal vascular development, cerebral vascular remodeling, and the maintenance of the BBB [107] (Table 1). During retinal vascular development, microglia secrete VEGF-C to activate the Notch signaling pathway in ETC via VEGFR-3, finally promoting the anastomosis of sprouting endothelial cells, which is essential for the construction of the retinal vascular network [108]. In developmental cerebral vascular remodeling, although microglia do not induce the pruning of developing brain vessels, they are recruited to clear apoptotic endothelial cells during vascular pruning, supporting the proper formation of the brain vascular network [109]. Microglia can also regulate brain vascular structure and function through the PANX1-P2RY12 signaling pathway. In mice lacking microglia, capillary is dilated and blood flow velocity is increased [110]. Furthermore, microglia play a vital role in repairing BBB damage. They promote the reconnection of endothelial cells at the ends of injured brain vessels, aiding in the restoration of BBB integrity [111]. However, under pathological conditions, activated microglia release inflammatory factors, inhibiting the expression of endothelial cell junction proteins and transporters, increasing the expression of LAM, and consequently affecting BBB function by increasing its permeability [112] (Figure 2).

### 4.5. Regulation of CNS Vascular Development by Neural Activity

Neural activity plays a significant regulatory role in neurovascular remodeling (Table 1). In adult rats, increased neural activity significantly promotes angiogenesis in the cerebellum [113]. Similarly, electroshock-induced epilepsy enhances angiogenesis in the hippocampal region [114]. In adult mice with focal ischemia in the whisker barrel cortex, increased neural activity from whisker stimulation promotes angiogenesis in the affected region [115]. During critical postnatal developmental periods, reduced sensory input leads to decreased vascular density, branching, and endothelial cell proliferation in the primary somatosensory cortex [115]. Conversely, increased sensory stimulation results in increased vascular density and branching. However, some studies have observed an opposite phenotype. For example, excessive neural activity caused by physical exercise, chemically induced epilepsy, or continuous auditory stimulation reduces cerebral cortex angiogenesis and results in abnormal vascular networks [116]. These findings suggest that the impact of neural activity on brain angiogenesis is not always positive and may depend on factors such as the intensity and duration of neural activity.

**Table 1:** Neural regulation of CNS vascular development and function.

Neural Cell Type	Factors	Functions	References
Neural progenitor cells	VEGF	Guide the infiltration of new blood vessels from PNVP into the brain parenchyma	[62,91]
	Wnt	CNS vascular development, Glut1 expression, and BBB development and maintenance	[69]
	integrin αVβ8	Specific knockout of integrin αVβ8 in NPCs leads to abnormal brain vascular development and intracranial hemorrhage	[68]
Astrocytes	Shh	Promote the expression of tight junction proteins Occludin and Claudin-5 in CNS endothelial cells to maintain BBB function	[99]
	VEGF	Increase BBB permeability under inflammatory condition	[105]
	Angiopoietin Angiotensin	Promote the formation of tight junctions in endothelial cells, regulating BBB function	[100]
	Retinoic acid	Upregulate the expression of junction proteins and transporters, promoting BBB development	[101]
	Apolipoprotein E	Regulate the expression of MMP-9 and BBB function	[102]
	aquaporin-4 Kir 4.1	Maintain brain water and ion homeostasis	[103,104]
Microglia	VEGF-C	Promote the anastomosis of sprouting endothelial cells for the construction of the retinal vascular network	[108]
Neurons	VEGF	Specific deletion of VEGF signaling in retinal interneurons leads to abnormal retinal vascular development	[93]
	Nogo-A	Negatively regulate brain angiogenesis	[94]
	miR-132	Promote brain vascular development and BBB maintenance	[95]
	Sema3A	Inhibit vascular reconstruction and disrupts the blood-retina barrier (BRB) in ischemic retina	[97]
	Neural activity	Increased neural activity promotes angiogenesis, while excessive neural activity reduces cerebral cortex angiogenesis and abnormal vascular networks	[115,116,117]
		Increased glutamatergic neural activity regulates angiogenesis and maturation of the retinal vascular barrier	[118]

The precise mechanisms by which neural activity regulates angiogenesis are not entirely clear. One possible explanation is that neural activity directly stimulates neurons to release various substances, such as neurotrophic factors and neurotransmitters, which act on endothelial cells, influencing their proliferation, migration, and angiogenesis processes. Another possibility is that neural activity indirectly regulates endothelial cell function by affecting other cell types, such as astrocytes and microglia. Recent research indicates that increased glutamatergic neural activity during retinal vascular development can regulate angiogenesis and the maturation of the retinal vascular barrier through the endothelial Norrin/β-catenin signaling pathway [117]. This finding provides a new perspective for understanding how neural activity regulates angiogenesis.

Additionally, neural activity can regulate the permeability of the BBB. For instance, neural activity can increase the expression of MMP-9 in vascular endothelial cells, thereby promoting the transcytosis of insulin-like growth factor-1 (IGF-1) from the blood into the CNS [118]. The excitation of spinal sensory neurons can increase the expression and release of chemokines in vascular endothelial cells, recruiting Th17 cells to gather and cross the barrier into the CNS [119]. Neural activity can also influence the outward transport of substances across the BBB by regulating circadian genes in endothelial cells, thereby modulating the clearance of waste from the CNS [120].

## 5. Conclusions and Perspective

The CNS vascular network is highly complex and specialized, forming a barrier structure to ensure efficient energy supply and microenvironmental homeostasis. Abnormalities in CNS vascular development and function are closely related to many neurological diseases [17]. Tissue microenvironment plays a crucial role in the formation of the specialized vascular network and the establishment of endothelial characteristics. Therefore, studying the regulation of vascular development and function by the nervous system, as well as identifying CNS-specific molecules, is of great importance. Excitingly, recent studies have revealed the regulatory role of the nervous system on brain vasculature, and identified some specific molecules that regulate CNS vascular development and function. However, research on CNS vascular development and function is still in its early stages, and more CNS-specific molecular mechanisms remain to be discovered.

A key question is how various cells within the CNS microenvironment coordinate to regulate the development and function of brain vasculature and the BBB. Furthermore, whether different brain regions have specific characteristics in terms of BBB development and function? In terms of the specific characteristics, how does neural activity regulate brain vascular and BBB development? These key questions in CNS vascular development urgently need research. Currently, much work relies on fixed sections to study CNS vasculature, limiting our ability to observe the dynamic changes in brain vascular development and function. The zebrafish, as a model organism with the advantage of in vivo imaging, has a relatively simple brain vascular network and vast of transgenic zebrafish lines labeling different CNS cell types, providing an ideal model to dynamically observe how endothelial cells and their surrounding cells coordinate to regulate brain vascular development and function [121]. Furthermore, with technological advancements, we can create model animals that label important BBB proteins, such as eGFP-Claudin5 transgenic mice or zebrafish, to dynamically observe the regulation of BBB development and function by cellular interactions in vivo. This not only helps us understand the biological characteristics of the BBB in more depth but also serves as an important model for drug screening. Through the fast development of methods for single-cell transcriptomics, the different characteristics of endothelial and related cells in different brain regions can be deeply analyzed, which may provide a comprehensive view of brain vascular and BBB development. Moreover, the development of methods for manipulating neural activity, including chemogenetic and optogenetic, will greatly facilitate the studies of neural activity-regulated brain vasculature and BBB development.

In summary, research on CNS vascular development and function still has vast exploratory space. By deeply studying the regulatory mechanisms of the nervous system on vascular development and function, we will provide new insights and methods for the prevention and treatment of neurological diseases.
